# The contribution of lesion location to upper limb deficit after stroke

**DOI:** 10.1136/jnnp-2015-312738

**Published:** 2016-07-22

**Authors:** Chang-hyun Park, Nancy Kou, Nick S Ward

**Affiliations:** 1 Sobell Department of Motor Neuroscience, UCL Institute of Neurology, London, UK; 2 Department of Neurology, Ewha Medical Research Institute, Ewha Womans University School of Medicine, Seoul, Korea; 3 The National Hospital for Neurology and Neurosurgery, London, UK; 4 UCLPartners Centre for Neurorehabilitation, UCL Institute of Neurology, London, UK

**Keywords:** STROKE, MRI, ANATOMY, IMAGE ANALYSIS

## Abstract

**Background:**

Motor deficit after stroke is related to regional anatomical damage.

**Objective:**

To examine the influence of lesion location on upper limb motor deficit in chronic patients with stroke.

**Methods:**

Lesion likelihood maps were created from T1-weighted structural MRI in 33 chronic patients with stroke with either purely subcortical lesions (SC, n=19) or lesions extending to any of the cortical motor areas (CM, n=14). We estimated lesion likelihood maps over the whole brain and applied multivoxel pattern analysis to seek the contribution weight of lesion likelihood to upper limb motor deficit. Among 5 brain regions of interest, the brain region with the greatest contribution to motor deficit was determined for each subgroup.

**Results:**

The corticospinal tract was most likely to be damaged in both subgroups. However, while damage in the corticospinal tract was the best indicator of motor deficit in the SC patients, motor deficit in the CM patients was best explained by damage in brain areas activated during handgrip.

**Conclusions:**

Quantification of structural damage can add to models explaining motor outcome after stroke, but assessment of corticospinal tract damage alone is unlikely to be sufficient when considering patients with stroke with a wide range of lesion topography.

## Introduction

Poor upper limb motor function is a major contributor to reduced quality of life after stroke.^1^ Understanding the causes of motor deficit will help to identify therapeutic approaches for all.^2^ A major contributor to upper limb motor deficit is the initial severity of stroke, but a number of other factors appear to be important, not least of which is the infarct location.^3^ The optimal way of assessing this quantitatively has not been determined and so anatomical damage is not routinely used to predict recovery potential after stroke. Although quantification of corticospinal tract damage is currently popular for explaining variability in motor outcome,^4^ it has largely been applied in patients with subcortical infarcts. We assessed the contribution of damage to other motor-related brain regions by creating individual lesion likelihood maps in an automated way and using multivoxel pattern analysis to determine the relation between damage within a number of a priori brain regions of interest (ROIs) and upper limb motor deficit. We then specifically asked whether quantification of damage in the corticospinal tract (or rather in a more widespread motor network) was enough to account for the variability in upper limb motor deficit in patients with either subcortical or combined cortical/subcortical damage.

## Methods

Thirty-three patients with stroke with first ever stroke at least 6 months previously (age 57.15±9.69 years, 12 females) were included in the study (table 1). We have previously characterised corticospinal tract damage in these patients.^5^ Patients were a priori divided into two subgroups: those with infarcts involving only subcortical areas (SC patients) and those with combined cortical/subcortical infarcts involving cortical motor areas (CM patients). CM areas were defined as primary motor cortex, premotor cortex and supplementary motor area, according to both approved anatomical and connectivity-based suggestions for parcellation.^6–8^ Twenty-three age-matched healthy participants (50.70±13.39 years) who reported no history of neurological illness, psychiatric history, vascular disease or hypertension, were employed to derive maps of normal brain structure, which acted as our ROIs. Full written consent was obtained from all participants in accordance with the Declaration of Helsinki. The study was approved by the Joint Ethics Committee of the Institute of Neurology, UCL and National Hospital for Neurology and Neurosurgery, UCL Hospitals National Health Service Foundation Trust, London, UK.

**Table 1:** Demographic and clinical characteristics of patients in this study

					Motor performance				
Subgroup	No	Age (years)	Gender	Affected hand	ARAT (0–57)	GRIP (%)	MI-UL (0–100)	NHPT (%)	PC1 (a.u.)
CM patients	1	60	Male	Left	39	20.1	65	0.0	−0.3167
	2	59	Male	Left	21	50.3	73	0.0	−0.2984
	3	59	Male	Left	52	64.4	42	14.9	−0.1048
	4	66	Male	Right	35	81.0	65	39.0	0.0300
	5	62	Female	Left	52	111.7	93	60.6	0.4627
	6	56	Female	Right	36	44.0	77	9.0	−0.1647
	7	56	Female	Right	39	41.0	77	9.0	−0.1547
	8	50	Male	Right	57	68.0	100	60.0	0.3666
	9	40	Female	Left	52	58.0	93	25.5	0.1298
	10	40	Female	Left	55	60.0	93	30.0	0.1756
	11	42	Female	Left	45	65.0	91	21.0	0.0803
	12	57	Male	Right	57	84.0	84	71.0	0.4003
	13	50	Female	Left	28	41.3	66	8.2	−0.2796
	14	52	Female	Right	27	31.0	73	0.0	−0.3266
		53.50±8.23	Male:female=6:8	Left:right=8:6	42.5±12.1	58.6±23.6	78.0±15.7	24.9±24.2	
SC patients	1	77	Female	Right	38	57.2	77	9.0	−0.2138
	2	52	Male	Left	42	35.0	88	9.0	−0.1929
	3	46	Male	Right	57	106.2	100	104.5	0.3099
	4	62	Male	Left	36	31.0	72	8.0	−0.3143
	5	53	Female	Left	50	40.0	91	50.0	−0.0415
	6	63	Male	Right	57	91.0	100	77.9	0.2191
	7	58	Male	Left	57	88.2	100	87.0	0.2308
	8	51	Male	Right	45	104.0	92	31.0	0.0542
	9	45	Female	Left	45	51.0	85	35.0	−0.1006
	10	69	Male	Right	57	80.5	100	69.7	0.1770
	11	55	Female	Left	55	64.0	93	97.0	0.1476
	12	61	Male	Left	45	51.1	65	19.7	−0.2298
	13	75	Male	Left	57	96.6	100	73.7	0.2242
	14	66	Male	Left	57	63.4	92.5	98.2	0.1565
	15	58	Male	Left	57	93.3	100	74.5	0.2178
	16	80	Male	Left	26	56.3	74	4.0	−0.3028
	17	60	Male	Left	26	24.9	48	5.1	−0.5046
	18	51	Male	Right	57	64.2	100	89.6	0.1779
	19	55	Male	Left	19	97.7	100	51.3	−0.0146
		59.84±10.00	Male:female=15:4	Left:right=13:6	46.5±12.4	68.2±26.0	88.3±14.8	52.3±36.1	

ARAT, Action Research Arm Test; CM, infarcts affecting primary and secondary motor cortices; GRIP, grip strength of affected hand given as a % of less affected hand; MI-UL, Motricity Index (upper limb component); NHPT, Nine-Hole Peg Test score of affected side given as a % of less affected side; PC1, first principle component of the four motor test scores (given as normalised values, arbitrary units); SC, infarcts involving subcortical structures.

We defined five prespecified ‘normal’ ROIs from healthy participants or the template brain (see online supplementary methods for details):^1^ corticospinal tract (CST),^2^ cortical sensorimotor areas (SM),^3^ brain areas activated during simple hand grip in healthy participants (activated sensorimotor (aSM)),^4^ all grey matter (GM) and^5^ all white matter (WM). The ROIs defined from healthy participants or the template brain, were used to assess the likelihood of damage to different brain structure thought to be important for motor function.

10.1136/jnnp-2015-312738.supp1 Supplementary data

All patients underwent the following: (1) a structural T1-weighted high-resolution MRI from which an individual voxel-wise lesion likelihood map was generated using the ALI toolbox (see online supplementary methods).^9^ We were then able to quantify the likelihood of damage within each of the ROIs defined above in each patient. (2) Measurement of upper limb motor deficit using the Action Research Arm Test, Nine-Hole Peg Test, Motricity Index and grip strength. A single representative score was calculated using principal component analysis in order to minimise floor and ceiling effects of individual tests. These data were used to determine the likelihood that damage in each voxel contributed to upper limb motor deficit, using Bayesian inference for regression termed a ‘relevance vector machine’ (http://www.mlnl.cs.ucl.ac.uk/pronto/). Leave-one-subject-out cross-validation was performed and the weight with which each voxel contributed to the regression function was summarised as a weight map for each patient subgroup, where a more negative value indicates that a lesion in that voxel is more likely to be associated with greater upper limb motor deficit.

Last, we calculated the contribution weight of each ROI to upper limb motor deficit as the average of voxel-wise values over the ROI in the weight map (for each patient subgroup). The lesion load was compared between each pair of the five ROIs, using paired samples t tests for each patient subgroup, and between the two patient subgroups, using two samples t tests for each ROI. Statistical significance was determined at a false discovery rate adjusted p value of 0.05. Note that statistical inferences for the weight maps were not feasible, as a weight map was acquired for each patient subgroup, not for each patient.

## Results

**Figure 1:**
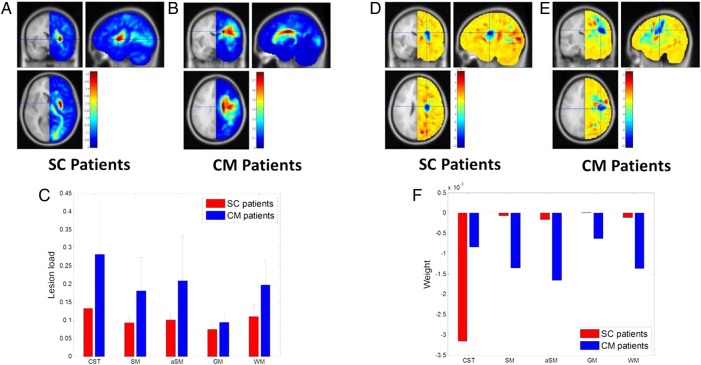
Lesion likelihood maps averaged across patients with subcortical lesions (SC patients) (A) and patients with lesions extending to cortical motor areas (CM patients) (B). In each map, the cross indicates the location of the most probable damage: x=28.5 mm, y=0.0 mm, z=4.5 mm in (A) and x=19.5 mm, y=−9.0 mm, z=28.5 mm in (B) in terms of Montreal Neurological Institute (MNI) coordinates. Comparison of lesion loads between brain regions of interest (ROIs) in SC patients and CM patients (C). Weight maps of SC patients (D) and CM patients (E). In each map, the cross indicates the location of the highest negative weight reflecting the greatest contribution of damage to upper limb motor deficit: x=25.5 mm, y=−9.0 mm, z=24.0 mm in (C) and x=43.5 mm, y=−16.5 mm, z=30.0 mm in (D) in terms of MNI coordinates. Comparison of contribution weights between ROIs in SC patients and CM patients (F). CST, SM, aSM, GM, and WM are ROI labels. For details about ROIs, refer to Online supplementary methods. CST, corticospinal tract; GM, grey matter; WM, white matter.

There were no significant differences between CM patients and SC patients in terms of age, gender or affected side. There was no difference in motor function between the two groups, apart from slower Nine-Hole Peg Test times for CM patients compared to those for SC patients (52.3±36.1 s vs 24.9±24.2 s respectively, p=0.02). Lesion likelihood maps for the two patient subgroups are shown in figure 1A, B. CM patients had significantly higher lesion loads than SC patients did, for all ROIs (p value=0.0009 for CST, p value=0.0002 for SM, p value=0.0009 for aSM, p value=0.0160 for GM and p value=0.0001 for WM) (figure 1C). Of the five ROIs, CST showed the greatest lesion load in both patient subgroups. In SC patients, the lesion load was greater in CST than in SM (p value=0.0339) and in GM (p value=0.0014). In CM patients, the lesion load was greater in CST than in GM (p value=0.0005). The full list of comparisons is provided in online supplementary tables S1 and S2.

Weight maps for the two patient subgroups are shown in figure 1D, E. The contribution towards upper limb motor deficit was the greatest (more negative value) in CST for SC patients and in aSM for CM patients (figure 1F). In SC patients, the contribution weight of CST to upper limb motor deficit was clearly dominant among the five ROIs, whereas in CM patients, the contribution weights of the five ROIs to upper limb motor deficit were comparable.

## Discussion

In this study, we examined the contribution to upper limb impairment made by damage in a set of brain regions in two groups of chronic patients with stroke with different lesion types. We showed that (1) the CST was the region most likely to be affected by both, subcortical and combined cortical/subcortical infarcts, and (2) CST damage was the most significant contributor to upper limb impairment in patients with subcortical infarcts, but not in those with combined cortical/subcortical infarcts. Although this result may be expected, it is important when considering how to use brain imaging to predict future motor outcomes after stroke.

The brain regions that contributed most to upper limb motor deficit in patients with subcortical or cortical/subcortical damage, respectively, correspond to the individual findings of previous lesion load studies.^10^ ^11^ However, our study involved several different methodological approaches. First, while these studies examined for lesions within only one ROI of either aSM^11^ or CST,^10^ we measured and compared the contribution towards upper limb motor deficit of damage in five different ROIs. Second, we estimated probable damage as a voxel-wise lesion likelihood map over the whole brain. These weighted values (ie, likelihood of lesion in each voxel) may be more useful than binary values (‘lesion’ or ‘not lesion’ in each voxel) given the increase in data points, but these two approaches have not yet been directly compared and so empirical data are required to determine the relative utility of each in predictive models in future. Third, previous studies have used univariate approaches such as voxel-based morphometry^12^ and voxel-based lesion-symptom mapping^13^ to assess the voxel-wise contribution of damage to functional deficits. Whereas we applied a multivariate approach—multivoxel pattern analysis—which allowed us to evaluate the relationship between distributed topographic patterns of brain damage and functional deficit rather than simply the contribution of damage in a single voxel. The current finding obtained with the multivariate approach verified the critical brain regions identified in terms of the voxel-wise contribution of damage to upper limb motor deficit with univariate approaches^14^ and, moreover, provided further insights into the influence of lesion location by revealing the ROI-wise contribution of damage to upper limb motor deficit.

In this contribution, we suggest that quantification of anatomical damage could be valuable in future attempts to reliably predict post-stroke motor outcome. However, although quantifying corticospinal damage is a useful first step toward predicting motor deficit, it may be insufficient if the aim is to develop a single automated process applicable to all patients, especially those with additional cortical damage. There are potential limitations in our approach. First, we acknowledge that our separation of patients based on infarct location is, on the face of it, arbitrary. However, the key point is that whether this a priori separation was optimal or not, it allowed us to answer a specific experimental question and to demonstrate a difference in the relationship between the location of stroke-related damage and motor deficit in the two patient subgroups. It is notable that dexterity (but not other motor scores) was more impaired in patients with additional cortical infarcts, indicating a possible concomitant aspect of different lesion topography. Second, we have looked for a relationship between motor deficit and lesion characteristics in a cross-sectional study. The characteristics of lesions may have changed over time, so that the use of brain images acquired in the early post-stroke phase to predict late outcome will need to be explicitly tested. Third, healthy participants from whose brains we derived the ROIs were not perfectly age-matched with patients. However, healthy participants were included to construct ROIs, and not to be directly compared to patients in the current study. Nevertheless, the importance of selecting the most relevant regions for assessing clinically relevant damage is clear. The five ROIs suggested in this study were hypothetically specified because of their potential for influencing motor deficit. However, in future, systematic approaches for selecting the most informative set of brain regions for predicting motor deficit will need to be performed.^15^

In summary, we have demonstrated that the brain regions that contribute most to upper limb motor deficit are different depending on lesion location and that one approach does not fit all. We suggest that the anatomy of damage should be considered in models for predicting motor outcome after stroke, but our study also illustrates that these models need to include a wider range of specified brain regions in order to be relevant for a broad range of stroke types.

## References

[1] Baker K, Barrett L, Playford ED, et al Measuring arm function early after stroke: is the DASH good enough? J Neurol Neurosurg Psychiatry 2016;87:604–10. 10.1136/jnnp-2015-310557 26180212

[2] Knecht S, Roßmüller J, Unrath M, et al Old benefit as much as young patients with stroke from high-intensity neurorehabilitation: cohort analysis. J Neurol Neurosurg Psychiatry 2016;87:526–30. 10.1136/jnnp-2015-310344 26069298 PMC4853552

[3] Coupar F, Pollock A, Rowe P, et al Predictors of upper limb recovery after stroke: a systematic review and meta-analysis. Clin Rehabil 2012;26:291–313. 10.1177/0269215511420305 22023891

[4] Jang SH A review of diffusion tensor imaging studies on motor recovery mechanisms in stroke patients. NeuroRehabilitation 2011;28:345–52. 10.3233/NRE-2011-0662 21725167

[5] Kou N, Park CH, Seghier ML, et al Can fully automated detection of corticospinal tract damage be used in stroke patients? Neurology 2013;80:2242–5. 10.1212/WNL.0b013e318296e977 23658388 PMC3721100

[6] Geyer S, Ledberg A, Schleicher A, et al Two different areas within the primary motor cortex of man. Nature 1996;382:805–7. 10.1038/382805a0 8752272

[7] Amiez C, Kostopoulos P, Champod AS, et al Local morphology predicts functional organization of the dorsal premotor region in the human brain. J Neurosci 2006;26:2724–31. 10.1523/JNEUROSCI.4739-05.2006 16525051 PMC6675158

[8] Nachev P, Kennard C, Husain M Functional role of the supplementary and pre-supplementary motor areas. Nat Rev Neurosci 2008;9:856–69. 10.1038/nrn2478 18843271

[9] Seghier ML, Ramlackhansingh A, Crinion J, et al Lesion identification using unified segmentation-normalisation models and fuzzy clustering. NeuroImage 2008;41:1253–66. 10.1016/j.neuroimage.2008.03.028 18482850 PMC2724121

[10] Zhu LL, Lindenberg R, Alexander MP, et al Lesion load of the corticospinal tract predicts motor impairment in chronic stroke. Stroke 2010;41:910–15. 10.1161/STROKEAHA.109.577023 20378864 PMC2886713

[11] Crafton KR, Mark AN, Cramer SC Improved understanding of cortical injury by incorporating measures of functional anatomy*.* Brain 2003;126:1650–9. 10.1093/brain/awg159 12805118

[12] Ashburner J, Friston KJ Voxel-based morphometry–the methods. NeuroImage 2000;11:805–21. 10.1006/nimg.2000.0582 10860804

[13] Bates E, Wilson SM, Saygin AP, et al Voxel-based lesion-symptom mapping. Nat Neurosci 2003;6:448–50. 10.1038/nn1050 12704393

[14] Lo R, Gitelman D, Levy R, et al Identification of critical areas for motor function recovery in chronic stroke subjects using voxel-based lesion symptom mapping. NeuroImage 2010;49:9–18. 10.1016/j.neuroimage.2009.08.044 19716427

[15] Forkert ND, Verleger T, Cheng B, et al Multiclass support vector machine-based lesion mapping predicts functional outcome in ischemic stroke patients. PloS ONE 2015;10:e0129569 10.1371/journal.pone.0129569 26098418 PMC4476759

