# Supplementary material for: The contribution of lesion location to upper limb deficit after stroke
Source: J Neurol Neurosurg Psychiatry. 2016 Jul 22;87(12):1283–6. doi: 10.1136/jnnp-2015-312738 (PMC5136717; doi:10.1136/jnnp-2015-312738)
Supplement: Supplementary data [file jnnp-2015-312738supp.pdf]

The contribution of lesion location to upper limb deficit after stroke.

Supplementary Methods

Patient selection

The patients in this study had been included in our previous work comparing manual and automated delineation of infarcts.[1] Here we included those with pure subcortical infarcts and infarcts involving both subcortical regions and one of primary motor cortex (M1), dorsal or ventral premotor cortex (PM) and supplementary motor area (SMA). Patients with purely cortical infarcts, those with cortical infarcts not involving any of primary motor cortex, dorsal or ventral premotor cortex and supplementary motor area, or those with brain stem infarcts were not included.

Assessment of motor function

The Action Research Arm Test, Nine-Hole Peg Test (NHPT), Motricity Index, and grip strength were assessed as previously described.[2] In particular, NHPT was performed by measuring the number of pegs placed per second with each hand, and individual patients' scores were obtained as the ratio of the impaired hand to the unimpaired hand. Grip strength was measured as the maximum strength for a given manipulandum, and individual patients' scores were determined as the ratio of the impaired hand to the unimpaired hand.

Principal component analysis of motor test scores

Four motor test scores were recorded per patient. For each motor test, motor test scores of patients in each subgroup were normalised to give unit variance and zero mean. Principal component analysis (PCA) was applied to the normalised scores of the four motor tests. Among four principal components extracted by PCA, the first principal component that explained the most variance across the four motor test scores was selected as representative scores for motor deficit of patients in the subgroup.

Acquisition of structural MRI data

In all subjects, structural magnetic resonance imaging (MRI) data were acquired as a T1-
weighted high resolution image (number of slices = 176, slice thickness = 1 mm, matrix size = 224 × 256, in-plane resolution = 1 mm × 1 mm).

Acquisition of fMRI data and DTI data in control subjects

As functional MRI (fMRI) data, a total of 120 images were acquired as T2*-weighted echo planar images during hand grip using the dominant hand (repetition time = 3,120 ms, echo time = 30 ms, number of slices = 48, slice thickness = 2 mm, slice gap = 1 mm, matrix size = 64 × 64, in-plane resolution = 3 mm × 3 mm). The onset and target force of each single hand grip was visually cued, where the target force was set to one of the prespecified ratios of each control's maximum voluntary contraction. More details on the motor task are described in our previous studies.[3, 4] As diffusion tensor imaging (DTI) data, a total of 68 images, including 61 images with high diffusion weighting (*b* value = 1000 s/mm^2) and 7 images with minimal diffusion weighting (*b* value = 100 s/mm^2), were acquired as T2-weighted echo planar images (number of slices = 60, slice thickness = 2.3 mm, matrix size = 96 × 96, in-plane resolution = 2.3 mm × 2.3 mm).

Lesion likelihood map

We generated a lesion likelihood map for each patient, as follows. The T1-weighted structural images were flipped about the mid-sagittal plane if lesions were in the left hemisphere. All structural images then showed lesions in the right hemisphere and were segmented into seven tissue types, including grey matter, white matter, cerebrospinal fluid, bone, soft tissue, air, and extra tissue, and then normalised using the New Segment routine in SPM8 (http://www.fil.ion.ucl.ac.uk/spm/). The extra tissue type corresponded to the area of infarcted brain and segmentation was repeated twice with an update of the extra tissue type to make sure segmented grey and white matter excluded the lesion. For each patient, the segmented grey and white matter was compared voxel by voxel with 23 healthy controls' segmented grey and white matter, so that the likelihood of damage in each voxel was calculated and expressed as a number between 0 and 1 using the algorithm in the ALI toolbox.[5]

Weight map

We then determined how much the likelihood of damage in each voxel contributed to upper limb motor deficit. Here, we employed a machine learning technique using Bayesian inference for regression termed a relevance vector machine.[6] This multi-voxel pattern analysis was performed in each patient subgroup using PRoNTo v1.1 (http://www.mlnl.cs.ucl.ac.uk/pronto/). The multivariate analysis enabled us to assess the association of lesion characteristics with upper limb motor deficit by considering the spatial correlation between all voxels over the grey and white matter, rather than regarding each voxel separately. Leave-one-subject-out cross-validation was performed and the weight with which each voxel contributed to the regression function was summarised as a weight map for each patient subgroup, where a more negative value indicates that a lesion in that voxel is more likely to be associated with greater upper limb motor deficit.

Brain regions of interest

The previous steps provided voxel-wise values of lesion likelihood (one lesion likelihood map per patient) and contribution to motor deficit (one weight map per patient subgroup). Next we were interested in assessing the importance of damage in a number of prespecified brain regions. Five brain regions of interest (ROIs) were defined from healthy controls or the template brain: (1) CST, (2) SM, (3) aSM, (4) GM, and (5) WM. CST was demarcated from healthy controls' DTI data by tracking the fibres from primary and secondary motor cortices including M1, PM, and SMA to ipsilateral lower pons via internal capsule and upper pons as previously described.[1] SM included M1, PM, and SMA as well as primary sensory cortex (S1) as defined in the Harvard-Oxford Atlas. aSM was acquired from healthy controls' fMRI data by determining group level activation during hand grip within SM at an uncorrected p value of 0.001. GM and WM were defined by keeping voxels with probabilities over 0.5 in the grey and white matter probability maps respectively included in SPM8. Note that GM includes basal ganglia and thalamus. More details about defining CST and aSM through analysis of fMRI and DTI data, respectively, are presented below.

Analysis of fMRI data

Preprocessing and statistical analysis of fMRI data were performed using SPM8. The first 6 images were discarded to allow for T1 equilibration effects, so that 114 images were used in preprocessing and statistical analysis. Each subject's 114 images were spatially realigned to the mean image and then unwarped to correct for head motions. The realigned images were normalised into the same coordinate frame as the MNI template brain with transformation parameters derived from segmentation of the subject's high resolution structural image coregistered to the mean functional image. The normalised images were spatially smoothed

using a Gaussian filter of 4 mm FWHM.
In first level statistical analysis of the preprocessed images, the general linear model included three regressors: (1) hand grip trials modelled as the delta function; (2) an interaction of hand grip with force exerted for it, modelled as the delta function scaled by the peak force; (3) a mean over all images. Each individual subject's activation map for the hand grip task was collected as voxel-wise parameter estimates of the first regressor. In group-level statistical analysis, a group-wise activation map for the hand grip task was acquired by carrying out a one sample *t* test of all subjects' activation maps. Finally, the group-level activations were thresholded at an uncorrected *p* value of 0.001 and were confined within sensorimotor cortex including M1, PM, SMA, and S1 to yield a brain region corresponding to healthy controls' activation map.

Analysis of DTI data
Preprocessing, diffusion tensor modelling, and tracking of corticospinal tract were performed using FDT v2.0 included in FSL (http://fsl.fmrib.ox.ac.uk/fsl/). Each subject's 68 images were first realigned to the first image to correct for eddy current-induced distortions and head motions. By Markov Chain Monte Carlo sampling, distributions of voxel-wise principal diffusion directions were inferred.
In probabilistic tractography of corticospinal tract, one seed mask, two waypoint masks, one target mask, and one exclusion mask were employed to spatially confine fibres. The seed mask comprised M1, PM, and SMA. The posterior limb of internal capsule (PLIC) and upper and lower pons ipsilateral to the seed mask were manually delineated, of which PLIC and upper pons served as the waypoint masks and lower pons as the target mask. The PLIC mask was placed from the level of anterior commissure to the base of corona radiata, and the upper and lower pons masks were located to only include anterior pons. Corpus callosum and cerebellum were used as the exclusion mask to remove inter-hemispheric and cerebellar trajectories. By repetitively computing 5,000 streamlines starting from every voxel of the seed mask, a distribution of streamline locations from the seed mask to the target mask via the waypoint masks was estimated. In the connectivity distribution, each voxel had a streamline count that passed through the voxel. Each individual subject's corticospinal tract map was acquired as a binary map by thresholding the subject's connectivity distribution at 5% of the maximum voxel value. A group-wise corticospinal tract map was generated by superposing all subjects' corticospinal tract maps and a brain region corresponding to healthy controls' corticospinal tract was determined to only include voxels with overlap counts higher than half of the subject number.

Lesion load and contribution weight of ROIs
Lastly, we wanted to calculate the following: (1) the lesion load of each ROI as the average of voxel-wise values over the ROI in the lesion likelihood map (for each patient) and (2) the contribution weight of each ROI to upper limb motor deficit as the average of voxel-wise values over the ROI in the weight map (for each patient subgroup). The lesion load was compared between each pair of the five ROIs using paired samples *t* tests for each patient subgroup, and between the two patient subgroup using two samples *t* tests for each ROI. Statistical significance was determined at a false discovery rate adjusted *p* value of 0.05. Also, the ROI with the greatest contribution weight to upper limb motor deficit was identified in each patient subgroup. Note that statistical inferences for the weight maps were not feasible, as a weight map was acquired for each patient subgroup, not for each patient.

Supplementary Tables

Table S1. Comparison of lesion loads between every pair of brain regions of interest (ROIs) in patients with infarcts only involving subcortical areas (SC-patients) (A) and patients with infarcts involving any of cortical motor areas (CM-patients) (B). Significant differences are indicated in red. CST, SM, aSM, GM, and WM are ROI labels. For details about ROIs, refer to Supplementary Methods.

(A) SC-patients

	CST	SM	aSM	GM	WM
CST		CST > SM (*p* value = 0.0339)	(*p* value = 0.0674)	CST > GM (*p* value = 0.0014)	(*p* value = 0.9057)
SM			(*p* value = 0.2307)	SM > GM (*p* value < 0.0001)	SM < WM (*p* value < 0.0001)
aSM				aSM > GM (*p* value < 0.0001)	aSM < WM (*p* value < 0.0001)
GM					GM < WM (*p* value < 0.0001)
WM					

(B) CM-patients

	CST	SM	aSM	GM	WM
CST		(*p* value = 0.2672)	(*p* value = 0.7423)	CST > GM (*p* value = 0.0005)	(*p* value = 0.1348)
SM			SM < aSM (*p* value = 0.0005)	SM > GM (*p* value = 0.0028)	(*p* value = 0.1804)
aSM				aSM > GM (*p* value < 0.0042)	(*p* value = 0.8197)
GM					GM < WM (*p* value < 0.0001)
WM					

Table S2. Comparison of lesion loads between patients with infarcts only involving

subcortical areas (SC-patients) and patients with infarcts involving any of cortical motor areas (CM-patients) for each brain region of interest (ROI). Significant differences are indicated in red. CST, SM, aSM, GM, and WM are ROI labels. For details about ROIs, refer to Supplementary Methods.

	CST	SM	aSM	GM	WM
SC-patients	0.1320	0.0929	0.1007	0.0738	0.1097
CM-patients	0.2810	0.1813	0.2080	0.0944	0.1970
SC-patients vs. CM-patients	SC < CM (*p* value = 0.0009)	SC < CM (*p* value = 0.0002)	SC < CM (*p* value = 0.0009)	SC < CM (*p* value = 0.0160)	SC < CM (*p* value < 0.0001)

References

1. Kou N, Park C, Seghier ML, Leff AP, Ward NS. Can fully automated detection of corticospinal tract damage be used in stroke patients? *Neurology*. 2013;80:2242-2245
2. Ward NS, Brown MM, Thompson AJ, Frackowiak RSJ. Neural correlates of motor recovery after stroke: A longitudinal fmri study. *Brain*. 2003;126:2476-2496
3. Park C, Boudrias M-H, Rossiter H, Ward NS. Age-related changes in the topological architecture of the brain during hand grip. *Neurobiology of aging*. 2012;33:833. e827-833. e837
4. Boudrias M-H, Gonçalves CS, Penny WD, Park C, Rossiter HE, Talelli P, et al. Age-related changes in causal interactions between cortical motor regions during hand grip. *Neuroimage*. 2012;59:3398-3405
5. Seghier ML, Ramlackhansingh A, Crinion J, Leff AP, Price CJ. Lesion identification using unified segmentation-normalisation models and fuzzy clustering. *NeuroImage*. 2008;41:1253-1266
6. Tipping ME. Sparse bayesian learning and the relevance vector machine. *Journal of Machine Learning Research*. 2001;1:211-244
